# A review of the mosquito species (Diptera: Culicidae) of Bangladesh

**DOI:** 10.1186/s13071-016-1848-z

**Published:** 2016-10-22

**Authors:** Seth R. Irish, Hasan Mohammad Al-Amin, Mohammad Shafiul Alam, Ralph E. Harbach

**Affiliations:** 1 Entomology Branch, Division of Parasitic Diseases and Malaria, Center for Global Health, Centers for Disease Control and Prevention, 1600 Clifton Rd NE, Atlanta, GA 30329 USA; 2 International Centre for Diarrhoeal Disease Research Bangladesh (icddr,b), 68 Shaheed Tajuddin Ahmed Sarani, Mohakhali, Dhaka 1212 Bangladesh; 3 Department of Life Sciences, Natural History Museum, Cromwell Road, London, SW7 5BD UK

**Keywords:** Species list, Mosquitoes, Bangladesh, Culicidae

## Abstract

**Background:**

Diseases caused by mosquito-borne pathogens remain an important source of morbidity and mortality in Bangladesh. To better control the vectors that transmit the agents of disease, and hence the diseases they cause, and to appreciate the diversity of the family Culicidae, it is important to have an up-to-date list of the species present in the country. Original records were collected from a literature review to compile a list of the species recorded in Bangladesh.

**Results:**

Records for 123 species were collected, although some species had only a single record. This is an increase of ten species over the most recent complete list, compiled nearly 30 years ago. Collection records of three additional species are included here: *Anopheles pseudowillmori*, *Armigeres malayi* and *Mimomyia luzonensis.*

**Conclusions:**

While this work constitutes the most complete list of mosquito species collected in Bangladesh, further work is needed to refine this list and understand the distributions of those species within the country. Improved morphological and molecular methods of identification will allow the refinement of this list in years to come.

## Background

Several diseases in Bangladesh are caused by mosquito-borne pathogens. Malaria remains an important cause of morbidity and mortality, particularly in the Chittagong Hill tracts and in the border belt areas [1]. Lymphatic filariasis continues to be a problem, despite multiple rounds of mass drug administration [2]. Dengue, Japanese encephalitis, and chikungunya viruses are also transmitted by mosquitoes in Bangladesh [3]. As knowledge of faunal diversity and vector control is often an important component of disease control, it is important to correctly identify and understand the bionomics of the species involved in transmission.

To this end, previous authors have worked to compile lists of the mosquito species present in Bangladesh, by compiling species records from the country. The mosquitoes of Bangladesh have been recorded in several such lists over the past century. James [4], Covell [5, 6], Christophers [7], Barraud [8] and Puri [9, 10] described the mosquito fauna of British India, from which some records for locations in present-day Bangladesh can be extracted. After the separation of Pakistan and India in 1947, Aslamkhan [11] published checklists for mosquito species, indicating which were found in East Pakistan (Bangladesh). After the independence of Bangladesh in 1971, Ahmed [12] published an updated checklist in 1987, including many new records. More recently, Bashar et al. [13] published a checklist for the *Anopheles* mosquitoes of Bangladesh, but no comprehensive list of all species of mosquitoes present in the country has been published for nearly 30 years. Over the decades, deforestation and increased agricultural practices may have altered the diversity and abundance of mosquitoes throughout the country [14–16]. Therefore, the aim of this work was to produce an updated list of all mosquito species reportedly present in Bangladesh, with information on their collection.

## Methods

PubMed and the US Armed Forces Pest Management Board Literature Retrieval Service (before it was terminated), were searched using the terms “mosquito” and “Bangladesh.” Articles found were searched for the descriptions of the original collections of mosquitoes identified to species. Articles only citing another article’s collections (second-hand accounts) were not included. The reference list of collected articles was searched for additional articles. National libraries of Bangladesh were also searched for articles. All articles were entered onto a spreadsheet to facilitate calculation of the number of species and the number of records for each species.

Additionally, one of us (HMA) provided records from studies that included the use of light traps (CDC miniature light trap, model 512, John W. Hock Co., Gainesville, Florida, USA), resting collections and BG-Sentinel traps (Biogents AG, Regensberg, Germany) (publications in preparation). The descriptions of the collection sites are provided in the discussion below.

## Results

Records were found for 123 species of Culicidae reported from Bangladesh. The records ranged in date from 1908 to 2016. These species are listed below, alphabetically by genus, subgenus, and species. *Aedes* species follow the classification provided by Wilkerson et al. [17], whereas all other genera follow the classification provided by the Mosquito Taxonomic Inventory [18]. In accordance with Article 51.3 of the *International Code of Zoological Nomenclature*, the authorship and date of species names enclosed in parentheses indicate that the species was originally described as a member of a genus other than the one in which it is currently placed, e.g. *Aedes aegypti* (Linnaeus, 1762) was originally described as *Culex aegypti* Linnaeus, 1762. The numbers following species names refer to articles where the records were published.

**Table:** 

**Genus** ***Aedeomyia*** **Theobald, 1901**
**Subgenus** ***Aedeomyia*** **Theobald, 1901**
1. *Ad.* (*Ady*.) *catastica* Knab, 1909 [11, 19]
**Genus** ***Aedes*** **Meigen, 1818**
**Subgenus** ***Aedimorphus*** **Theobald, 1903**
2. *Ae*. (*Adm*.) *caecus* (Theobald, 1901) [12, 20–22]
3. *Ae*. (*Adm*.) *pallidostriatus* (Theobald, 1907) [12, 20, 21]
4. *Ae*. (*Adm*.) *punctifemoris* (Ludlow, 1921) [12, 20–23]
5. *Ae.* (*Adm.*) *vexans* (Meigen, 1830) [12, 19, 24, 25]
**Subgenus** ***Christophersiomyia*** **Barraud, 1923**
6. *Ae*. (*Chr.*) *thomsoni* (Theobald, 1905) [21]
**Subgenus** ***Collessius*** **Reinert, Harbach & Kitching, 2006**
7. *Ae.* (*Col.*) *pseudotaeniatus* (Giles, 1901) [26]
**Subgenus** ***Dendroskusea*** **Edwards, 1929**
8. *Ae*. (*Dsk*.) *reginae* Edwards, 1922 [26]
**Subgenus** ***Downsiomyia*** **Vargas, 1950**
9. *Ae*. (*Dow.*) *albolateralis* (Theobald, 1908) [27]
10. *Ae*. (*Dow.*) *niveus* (Ludlow, 1903) [12, 24, 28, 29]
**Subgenus** ***Edwardsaedes*** **Belkin, 1962**
11. *Ae.* (*Edw.*) *imprimens* (Walker, 1861) [30]
**Subgenus** ***Fredwardsius*** **Reinert, 2000**
12. *Ae*. (*Fre*.) *vittatus* (Bigot, 1861) [26]
**Subgenus** ***Hulecoeteomyia*** **Theobald, 1904**
13. *Ae.* (*Hul.*) *chrysolineatus* (Theobald, 1907) [12, 20, 28, 31]
**Subgenus** ***Kenknightia*** **Reinert, 1990**
14. *Ae.* (*Ken.*) *dissimilis* (Leicester, 1908) [12, 28, 32]
**Subgenus** ***Mucidus*** **Theobald, 1901**
15. *Ae.* (*Muc*.) *scatophagoides* (Theobald, 1901) [12, 20, 21, 24, 27, 31, 33, 34]
**Subgenus** ***Neomelaniconion*** **Newstead, 1907**
16. *Ae*. (*Neo*.) *lineatopennis* (Ludlow, 1905) [12, 19–21, 24, 31, 35–37]
**Subgenus** ***Petermattinglyius*** **Reinert, Harbach & Kitching, 2009**
17. *Ae.* (*Pet.*) *iyengari* Edwards, 1923 [26]
**Subgenus** ***Phagomyia*** **Theobald, 1905**
18. *Ae.* (*Phg*.) *assamensis* (Theobald, 1908) [11, 12, 21, 24, 27–29, 38, 39]
19. *Ae.* (*Phg*.) *khazani* Edwards, 1922 [8, 12, 24, 26, 28, 29]
20. *Ae.* (*Phg*.) *lophoventralis* (Theobald, 1910) [11, 12, 28, 29, 36, 37]
**Subgenus** ***Stegomyia*** **Theobald, 1901**
21. *Ae*. (*Stg*.) *aegypti* (Linnaeus, 1762) [12, 25, 26, 28, 34, 36, 37, 40–54]
22. *Ae*. (*Stg*.) *albopictus* (Skuse, 1895) [12, 19–21, 24–28, 31, 36, 37, 40, 42, 44–46, 49, 50, 52–58]
23. *Ae*. (*Stg*.) *annandalei* (Theobald, 1910) [11, 12, 20, 26, 28]
24. *Ae*. (*Stg*.) *desmotes* (Giles, 1904) [59]
25. *Ae*. (*Stg*.) *gardnerii imitator* (Leicester, 1908) [60]
26. *Ae*. (*Stg*.) *w-albus* (Theobald, 1905) [12, 28]
**Genus** ***Anopheles*** **Meigen, 1818**
**Subgenus** ***Anopheles*** **Meigen, 1818**
27. *An*. (*Ano*.) *aitkenii* James, 1903 [12, 13, 21, 61–64]
28. *An*. (*Ano*.) *baileyi* Edwards, 1929 [7, 9, 63]
29. *An*. (*Ano*.) *barbirostris* van der Wulp, 1884 (*s.l*.) [5, 6, 9, 10, 12–16, 19–21, 24, 31, 34–37, 40, 47, 51, 61–82]
30. *An*. (*Ano*.) *barbumbrosus* Strickland & Chowdhury, 1927 [83]
31. *An*. (*Ano*.) *bengalensis* Puri, 1930 [12, 34]
32. *An*. (*Ano*.) *gigas* Giles, 1901 [6, 34, 63, 84]
33. *An*. (*Ano*.) *nigerrimus* Giles, 1900 [5, 6, 12–15, 19–21, 26, 34–37, 40, 47, 48, 61–64, 66, 68, 69, 72, 73, 82, 85]
34. *An*. (*Ano*.) *peditaeniatus* (Leicester, 1908) [12, 13, 15, 19, 20, 24, 31, 34, 41, 42, 47, 61–64, 66, 67, 71, 86]
35. *An*. (*Ano*.) *umbrosus* (Theobald, 1903) [5, 6, 13–15, 35, 61–64, 68]
**Subgenus** ***Cellia*** **Theobald, 1902**
36. *An.* (*Cel*.) *aconitus* Dönitz, 1902 [6, 9, 10, 12–16, 20, 21, 24, 31, 34, 36, 37, 48, 51, 63, 68, 70, 72, 73, 75–78, 80–82, 87, 88]
37. *An.* (*Cel*.) *annularis* van der Wulp, 1884 (*s.l*.) [5, 6, 10, 12–16, 19–21, 24, 25, 31, 34–37, 40–42, 45, 51, 61–65, 67–73, 75–78, 80–82, 86, 88]
38. *An.* (*Cel*.) *baimaii* Sallum & Peyton, 2005 [12–15, 21, 24, 61–64, 81–83, 87, 89–94]
39. *An.* (*Cel*.) *culicifacies* Giles, 1901 (*s.l*.) [5, 6, 10, 12–14, 16, 20, 21, 24, 25, 31, 34, 36, 37, 45, 63, 70, 72, 73, 87]
40. *An.* (*Cel*.) *fluviatilis* James, 1902 (*s.l*.) [5, 6, 12, 13, 24, 34, 48, 61–63, 87]
41. *An.* (*Cel*.) *jamesii* Theobald, 1901 [5, 6, 10, 12–16, 20, 24, 31, 34, 36, 37, 47, 48, 61–64, 67, 68, 70, 71, 73, 78, 81, 82]
42. *An.* (*Cel*.) *jeyporiensis* James, 1902 [5, 6, 10, 12–15, 20, 21, 24, 31, 61–64, 68, 70, 73, 78, 81, 82, 87]
43. *An.* (*Cel*.) *karwari* (James, 1903) [6, 9, 12–15, 20, 24, 35, 61–64, 68, 81, 82]
44. *An.* (*Cel*.) *kochi* Dönitz, 1901 [4–6, 10, 12–15, 24, 34, 35, 61–64, 68, 70, 71, 73, 81–83, 85, 87]
45. *An.* (*Cel*.) *maculatus* Theobald, 1901 [12–15, 24, 34, 61–64, 68, 81]
46. *An.* (*Cel*.) *majidi* Young & Majid, 1928 [12, 13, 24, 63]
47. *An.* (*Cel*.) *minimus* Theobald, 1901 (*s.l*.) [6, 12–15, 24, 61–64, 68, 70, 77, 78, 80, 82, 87, 95, 96]
48. *An.* (*Cel*.) *nivipes* (Theobald, 1903) [13–15, 62, 64]
49. *An.* (*Cel*.) *pallidus* Theobald, 1901 [6, 10, 12, 13, 16, 20, 24, 34, 61–64, 70, 72, 73, 77, 80]
50. *An.* (*Cel*.) *philippinensis* Ludlow, 1902 [5, 6, 10, 12, 13, 15, 16, 20, 24, 31, 34, 35, 61–64, 68, 70–73, 77, 78, 80–82, 87, 93, 95, 97, 98]
51 *An.* (*Cel*.) *pseudojamesi* Strickland & Chowdhury, 1927 [6, 10, 12, 13, 16, 35–37, 62–64, 70–73, 77, 78]
52. *An.* (*Cel*.) *pseudowillmori* (Theobald, 1910) [Al-Amin, in preparation]
53. *An.* (*Cel*.) *splendidus* Koidzumi, 1920 [6, 12, 13, 62, 63, 99]
54. *An.* (*Cel*.) *stephensi* Liston, 1901 [47, 100]
55. *An.* (*Cel*.) *subpictus* Grassi, 1899 (*s.l*.) [5, 6, 10, 12–16, 19–21, 24, 27, 31, 34–37, 40–42, 47, 48, 51, 61–72, 77–79, 81, 82, 87, 101]
56. *An.* (*Cel*.) *sundaicus* (Rodenwaldt, 1925) (*s.l*.) [6, 10, 12, 13, 16, 48, 63, 72, 73, 77, 79, 95]
57. *An.* (*Cel*.) *tessellatus* Theobald, 1901 [5, 6, 10, 12–16, 19, 20, 24, 34, 36, 37, 61–64, 68, 70, 72, 73, 81, 83]
58. *An.* (*Cel*.) *theobaldi* Giles, 1901 [5, 6]
59. *An.* (*Cel*.) *turkhudi* Liston, 1901 [14, 48]
60. *An.* (*Cel*.) *vagus* Dönitz, 1902 [6, 9, 10, 12–16, 19–21, 24–26, 31, 34–37, 40–42, 47, 48, 51, 61–72, 75–78, 80, 82, 86–88]
61. *An.* (*Cel*.) *varuna* Iyengar, 1924 [6, 10, 12–16, 19, 20, 24, 34, 48, 61–63, 68, 70, 72, 73, 77, 82, 87]
62. *An.* (*Cel*.) *willmori* (James, 1903) [6, 12–15, 24, 62–64, 68]
**Genus** ***Armigeres*** **Theobald, 1901**
**Subgenus** ***Armigeres*** **Theobald, 1901**
63. *Ar.* (*Arm.*) *kesseli* Ramalingam, 1987 [19]
64. *Ar.* (*Arm.*) *kuchingensis* Edwards, 1915 [12, 19, 20, 24, 28, 35–37, 41, 42, 47, 48]
65. *Ar.* (*Arm.*) *malayi* (Theobald, 1901) [Al-Amin, in preparation]
66. *Ar.* (*Arm.*) *subalbatus* (Coquillett, 1898) [12, 19–21, 24–28, 34, 36, 37, 40–42, 44, 45, 50, 51, 55, 65, 66, 86, 102]
67. *Ar.* (*Arm.*) *theobaldi* Barraud, 1934 [103]
**Subgenus** ***Leicesteria*** **Theobald, 1904**
68. *Ar.* (*Lei*.) *annulitarsis* (Leicester, 1908) [12]
69. *Ar.* (*Lei*.) *dentatus* Barraud, 1927 [12]
70. *Ar.* (*Lei*.) *digitatus* (Edwards, 1914) [12, 24, 31]
71. *Ar.* (*Lei*.) *flavus* (Leicester, 1908) [11, 12, 24, 34, 102]
72 *Ar.* (*Lei*.) *inchoatus* Barraud, 1927 [12]
73. *Ar.* (*Lei*.) *magnus* (Theobald, 1908) [11, 12, 20, 21, 24, 27, 102]
74. *Ar.* (*Lei*.) *omissus* (Edwards, 1914) [12]
**Genus** ***Coquillettidia*** **Dyar, 1905**
**Subgenus** ***Coquillettidia*** **Dyar, 1905**
75. *Cq.* (*Coq.*) *crassipes* (van der Wulp, 1881) [12, 19, 20, 24, 27, 31, 48, 104]
76. *Cq.* (*Coq.*) *ochracea* (Theobald, 1903) [12, 104]
**Genus** ***Culex*** **Linnaeus, 1758**
**Subgenus** ***Culex*** **Linnaeus, 1758**
77. *Cx.* (*Cux.*) *annulus* Theobald, 1901 [35, 47, 48]
78. *Cx.* (*Cux.*) *fuscocephala* Theobald, 1907 [12, 19–21, 24–26, 31, 35–37, 41, 42, 44, 45, 48, 50, 51, 65–67, 86, 105–108]
79. *Cx.* (*Cux.*) *gelidus* Theobald, 1901 [12, 19–21, 24, 25, 27, 31, 34–37, 40–42, 50, 51, 55, 65–67, 86, 106, 109]
80. *Cx.* (*Cux.*) *hutchinsoni* Barraud, 1924 [12, 19, 34, 35, 47, 48, 50]
81. *Cx.* (*Cux.*) *mimulus* Edwards, 1915 [12, 35, 66, 108, 109]
82. *Cx.* (*Cux.*) *pseudovishnui* Colless, 1957 [12, 19, 20, 34, 108]
83. *Cx.* (*Cux.*) *quinquefasciatus* Say, 1823 [12, 19–21, 24–28, 31, 34–37, 40–42, 44–48, 50, 51, 55, 57, 65–67, 108, 110–114]
84. *Cx.* (*Cux.*) *sitiens* Wiedemann, 1828 [27, 34, 47]
85. *Cx.* (*Cux.*) *theileri* Theobald, 1903 [21]
86. *Cx.* (*Cux.*) *tritaeniorhynchus* Giles, 1901 [12, 19–21, 24, 25, 31, 34–37, 40–42, 45, 50, 51, 55, 65–67, 86, 108, 109, 115]
87. *Cx.* (*Cux.*) *vagans* Wiedemann, 1828 [21]
88. *Cx.* (*Cux.*) *vishnui* Theobald, 1901 [12, 19, 20, 24–27, 31, 34, 35, 41, 42, 50, 65, 67, 83, 86, 108, 109]
89. *Cx.* (*Cux.*) *whitei* Barraud, 1923 [36, 37, 108]
90. *Cx.* (*Cux.*) *whitmorei* (Giles, 1904) [12, 19–21, 24, 31, 34–37, 86, 108]
**Subgenus** ***Culiciomyia*** **Theobald, 1907**
91. *Cx.* (*Cui.*) *fragilis* Ludlow, 1903
92. *Cx.* (*Cui.*) *nigropunctatus* Edwards, 1926 [8, 12]
93. *Cx.* (*Cui.*) *pallidothorax* Theobald, 1905 [12, 24, 26, 116]
94. *Cx.* (*Cui.*) *pullus* Theobald, 1905 [116]
**Subgenus** ***Eumelanomyia*** **Theobald, 1909**
95. *Cx.* (*Eum*.) *brevipalpis* (Giles, 1902) [12, 24, 26, 28, 117]
96*. Cx.* (*Eum*.) *malayi* (Leicester, 1908) [12, 24]
**Subgenus** ***Lophoceraomyia*** **Theobald, 1905**
97. *Cx.* (*Lop*.) *minutissimus* (Theobald, 1907) [12]
**Subgenus** ***Oculeomyia*** **Theobald, 1907**
98. *Cx.* (*Ocu*.) *bitaeniorhynchus* Giles, 1901 [12, 19–21, 24, 27, 31, 34–37, 41, 42, 51, 55, 65–67, 86, 108, 109]
99. *Cx.* (*Ocu*.) *epidesmus* (Theobald, 1910) [12, 19–21, 24, 31, 34, 35, 47, 48, 108]
100. *Cx.* (*Ocu*.) *infula* Theobald, 1901 [19, 27, 108, 118]
101. *Cx.* (*Ocu*.) *sinensis* Theobald, 1903 [12, 19–21, 24, 31, 34, 35, 66, 86]
**Genus** ***Ficalbia*** **Theobald, 1903**
102. *Fi. minima* (Theobald, 1901) [8, 12, 27, 33, 34, 36, 37, 119]
**Genus** ***Heizmannia*** **Ludlow, 1905**
**Subgenus** ***Heizmannia*** **Ludlow, 1905**
103. *Hz.* (*Hez*.) *covelli* Barraud, 1929 [26]
**Genus** ***Lutzia*** **Theobald, 1903**
**Subgenus** ***Metalutzia*** **Tanaka, 2003**
104. *Lt.* (*Mlt*.) *fuscana* (Wiedemann, 1820) [12, 19, 20, 24, 28, 31, 34, 36, 37, 41, 42, 120]
105. *Lt.* (*Mlt*.) *halifaxii* (Theobald, 1903) [12, 34, 83]
**Genus** ***Malaya*** **Leicester, 1908**
106. *Ml. genurostris* Leicester, 1908 [8, 12, 20, 24, 28, 121]
107. *Ml. jacobsoni* (Edwards, 1930) [8]
**Genus** ***Mansonia*** **Blanchard, 1901**
**Subgenus** ***Mansonioides*** **Theobald, 1907**
108. *Ma.* (*Mnd*.) *annulata* Leicester, 1908 [19]
109. *Ma.* (*Mnd*.) *annulifera* (Theobald, 1901) [11, 12, 19–21, 24, 25, 27, 31, 34–37, 41, 42, 45, 50, 65, 67, 104]
110. *Ma.* (*Mnd*.) *dives* (Schiner, 1868) [12, 104]
111. *Ma.* (*Mnd*.) *indiana* Edwards, 1930 [12, 19–21, 24, 31, 34, 35, 50, 65]
112. *Ma.* (*Mnd*.) *uniformis* (Theobald, 1901) [12, 19–21, 24, 25, 27, 31, 34–37, 41, 42, 45, 50, 55, 65, 67, 104]
**Genus** ***Mimomyia*** **Theobald, 1903**
**Subgenus** ***Etorleptiomyia*** **Theobald, 1904**
113. *Mi.* (*Eto.*) *luzonensis* (Ludlow, 1905) [Al-Amin, in preparation]
**Subgenus** ***Mimomyia*** **Theobald, 1903**
114. *Mi.* (*Mim*.) *chamberlaini* Ludlow, 1904 [12, 33, 40, 122]
115. *Mi.* (*Mim*.) *hybrida* (Leicester, 1908) [8, 119]
**Genus** ***Orthopodomyia*** **Theobald, 1904**
116. *Or. anopheloides* (Giles, 1903) [26, 123, 124]
**Genus** ***Toxorhynchites*** **Theobald, 1901**
**Subgenus** ***Toxorhynchites*** **Theobald, 1901**
117. *Tx.* (*Tox*.) *bengalensis* Rosenberg & Evenhuis, 1985 [125]
118. *Tx.* (*Tox*.) *splendens* (Wiedemann, 1819) [12, 26, 28, 36, 45, 126, 127]
**Genus** ***Tripteroides*** **Giles, 1904**
**Subgenus** ***Rachionotomyia*** **Theobald, 1905**
119. *Tp.* (*Rah*.) *aranoides* (Theobald, 1901) [12, 21, 24, 28, 47]
**Genus** ***Uranotaenia*** **Lynch Arribálzaga, 1891**
**Subgenus** ***Pseudoficalbia*** **Theobald, 1912**
120. *Ur.* (*Pfc*.) *novobscura* Barraud, 1934 [12]
**Subgenus** ***Uranotaenia*** **Lynch Arribálzaga, 1891**
121. *Ur.* (*Ura*.) *campestris* Leicester, 1908 [12, 20]
122. *Ur.* (*Ura*.) *rampae* Peyton & Klein, 1970 [19]
**Genus** ***Verrallina*** **Theobald, 1903**
**Subgenus** ***Neomacleaya*** **Theobald, 1907**
123. *Ve.* (*Nma*.) *andamanensis* (Edwards, 1922) [8, 27, 128]

## Discussion

A need for improved knowledge of the mosquito species present in Bangladesh has led to the updated list presented here. In some cases previously known species have been found to be species complexes, while in other cases the names of species have been changed or subspecies elevated to species status. In several cases, new collections have resulted in species representing new country records being added to the list.

Among the listed mosquitoes, *Anopheles* species complexes that are worthy of note here include the Subpictus, Culicifacies, Sundaicus, Annularis, Barbirostris, Fluviatilis, Maculatus, Minimus, and Dirus Complexes. The Subpictus Complex includes four sibling species (informally designated species A, B, C, and D), which are distinguishable based on morphological, chromosomal, and molecular differences [129, 130]. As of yet, no determination has been made of which species of the Subpictus Complex are present in Bangladesh. *Anopheles culicifacies* is an important malaria vector in the Indian Sub-continent, but its importance in Bangladesh appears to be limited. The Culicifacies Complex is comprised of species A, B, C, D, and E [131]. Little is known of the species present in Bangladesh, particularly as very few specimens have been found in the past 20 years [22, 63], but recently species B, C, and E were found in Kuhalong, near Bandarban town [14]. The Sundaicus Complex comprises four coastal species: *An. sundaicus*, *An. epiroticus*, *An. sundaicus* D, and *An. sundaicus* E [132]. While it would appear that *An. epiroticus* is most likely the species present in coastal areas of Bangladesh, due to the limited geographic provenance of the other species, this awaits confirmation using molecular methods [132]. *Anopheles annularis* is a complex of two species, A and B [133], which can be distinguished using chromosomal or molecular methods [134]. The Fluviatilis Complex is composed of four sibling species, S, T, U, and V [135], but it is not known which species are present in Bangladesh. The Maculatus Complex (a.k.a. Maculatus Group) consists of nine species, but *Anopheles maculatus* is comprised of chromosomal forms B and E [136, 137]. Rongnoparut et al. [138] stated that chromosomal form B is found north of 13°N, but further work on the group is necessary to know which members are present in Bangladesh. The Barbirostris Complex is composed of seven formally named species, but it seems more species will be added [139, 140]. Only *An. barbirostris * (*s.l*.) has been reported from Bangladesh, but it is clear that more work is needed to know which species of the complex are present in the country. The Minimus Complex includes three species: *An. harrisoni*, *An. minimus* and *An. yaeyamaensis* (known only from Japan). *Anopheles minimus* (*s.l*.) has been found in Bangladesh [14], but further work is needed to know if *An. harrisoni* (previously known as species C) is present in the country [141]. Finally, the Dirus Complex comprises seven species, of which it appears *An. baimaii* is the species present in Bangladesh [14, 92, 142].

Some of the species included in the most recent previous list published by Ahmed [12] have undergone taxonomic changes. *Anopheles gigas* var. *baileyi* was elevated to species status by Harrison et al. [143] and is now known as *Anopheles baileyi*. Similarly, *Anopheles maculatus* var. *willmori* (as *willmorei*) was restored to species status by Rattanarithikul & Green [144] and is now known as *An. willmori. Anopheles pseudojamesi* is the current name (senior synonym) of the formerly accepted *An. ramsayi* [79]. *Culex afridi* was synonymized with *Cx. infula* [109]. *Culex quinquefasciatus* was listed as “*Culex pipiens quinquefasciatus*” in the list provided by Ahmed, but *Cx. quinquefasciatus* is now accepted as a distinct species [145, 146]. The species formerly known as *Cx. fuscanus* and *Cx. halifaxii* are now included in the genus *Lutzia* as *Lt. fuscana* and *Lt. halifaxii*, respectively [147].

There are a number of species that have only been reported in Bangladesh since the list of Ahmed [12]. Larvae of *Ae. gardnerii imitator* were collected from a tree hole in a botanical garden in Dhaka during 2004 [60]. Adults of *Ar. kesseli* were taken in resting collections and light traps in Rajshahi Division [19], and adults of *Ar. theobaldi* were collected in a forested area in Habiganj district (Sylhet Division) [103]. *Culex annulus* has been collected in Habiganj and Moulavibazar districts in Sylhet Division, as well as on St. Martin’s Island, and in the Sundarbans [35, 47, 48]. Two adults of *Ma. annulata* and three of *Ur. rampae* were collected in light traps in Rajshahi Division [19]. *Culex fragilis* was collected in resting catches in Patrokhola tea garden, Moulavibazar (Sylhet Division) [35]. Additionally, one of us (HMA) recently identified three species new to Bangladesh, which will be reported in publications currently in preparation. *Anopheles pseudowillmori* were collected in light traps placed in a room with sleeping people (18:00 to 06:00). Nine specimens were collected in Kuhalong and Rajbila unions of Bandarban district between 2009 and 2012. Three *Ar. malayi* females were collected in BG-Sentinel traps that were placed indoors in Rajshahi and Chapai Nawabganj districts between November 2014 and September 2015. *Mimomyia luzonensis* was collected using the same method in Dhaka and Chapai Nawabganj districts, and four specimens were collected between September 2014 and September 2015.

Two species listed in Ahmed [12] are excluded from the list. Ahmed [12] listed *Ae. pipersalatus* with reference to Barraud [30]. In the older reference, a record is provided for Madhupur in Bengal, attributed to Charles Paiva, an entomologist educated in Calcutta, who then took up a position at the Indian Museum in Calcutta where he worked until his death in 1919 [148]. There is a sub-district called Madhupur near Dhaka in Bangladesh, however, there is also a Madhupur in West Bengal in India. As we could not find any further evidence to which Madhupur was intended, and as no further records of *Ae. pipersalatus* in Bangladesh were found, we have removed this species from the list of species known to occur in the country. We have also removed the separate listing of *An. gigas* subspecies *simlensis* [149], which has been reported only once, from Rangpur [6], as it is represented by *An. gigas* in the species list.

*Armigeres obturbans* has been listed in several records, but it appears that this species was regularly confused with the common *Ar. subalbatus*. Furthermore, Thurman [150] noted that the distribution of *Ar. obturbans* is limited to Sulawesi, so it seems likely that the records for *Ar. obturbans* in Bangladesh refer to *Ar. subalbatus*. We have therefore removed *Ar. obturbans* from the species list for Bangladesh.

The inclusion of certain species in previous lists was the result of single records. This was the case for *Ae. thomsoni*, *Ae. pseudotaeniatus*, *Ae. reginae*, *Ae. albolateralis*, *Ae. imprimens*, *Ae. vittatus*, *Ae. iyengari*, *Ae. desmotes*, *Ae. gardnerii imitator*, *An. barbumbrosus*, *Ar. kesseli*, *Ar. theobaldi*, *Ar. annulitarsis*, *Ar. dentatus*, *Ar. inchoatus*, *Ar. omissus*, *Cx. fragilis*, *Cx. theileri*, *Cx. vagans*, *Cx. pullus*, *Cx. minutissimus*, *Hz. covelli*, *Ml. jacobsoni*, *Ma. annulata*, *Tx. bengalensis*, *Ur. novobscura*, and *Ur. rampae*. While several of these species represent recent records for Bangladesh, others, such as *Ae. pseudotaeniatus*, *Ae. reginae*, *Ae. albolateralis*, *Ae. imprimens*, *Ae. vittatus*, *Ae. iyengari*, *Cx. pullus*, *Hz. covelli*, and *Ml. jacobsoni*, have not been found in Bangladesh in the past 50 years. Further collections, particularly of culicines, would be helpful in improving our knowledge of the species that are present in the country.

## Conclusions

The list provided here is the most complete list compiled since the work of Ahmed [12], nearly 30 years ago. The up-dated list will serve as a basis for the development of identification keys and will hopefully spur on research on the bionomics of these species. These tools will be important for the reduction of diseases in Bangladesh caused by mosquito-borne pathogens.
